# Negative effects of heat stress on ovarian tissue in female rabbit

**DOI:** 10.3389/fvets.2022.1009182

**Published:** 2022-11-14

**Authors:** Lipeng Tang, Xue Bai, Xiaohong Xie, Guanhe Chen, Xianbo Jia, Ming Lei, Congyan Li, Songjia Lai

**Affiliations:** ^1^College of Animal Science and Technology, Sichuan Agricultural University, Chengdu, China; ^2^Sichuan Provincial Key Laboratory of Animal Genetics and Breeding, Sichuan Academy of Animal Science, Chengdu, China

**Keywords:** heat stress, rabbit, ovary, microRNA, gene expression

## Abstract

Numerous studies have highlighted the role of miRNA in the deformation and necrosis of cells of ovarian tissue caused by heat stress (HS), which ultimately affects ovarian function. Although the role of small RNAs has been investigated in alterations in ovarian tissue functioning in response to HS, the expression profile of ovarian miRNA has been explored to a lesser extent. In this study, female rabbits were subject to HS treatment by using electrical heater. The current work demonstrated that HS could significantly change physiological performance of female rabbits including body weight, rectal temperature and relative ovary weight, and significantly reduce serum *IL-2, IL-8, CAT*, and *GSH-Px* concentrations by enzyme-linked immunosorbent assay (ELISA) technique. As a result, an increase in apoptosis in ovarian cells, as well as unhealthy follicles, were observed by Hematoxylin-eosin (HE) and TUNEL staining. Additionally, small RNA-seq revealed changes in the miRNA expression profile of rabbit ovaries under HS. Five hundred fourteen miRNAs were obtained including known miRNAs 442 and novel miRNAs 72. Among these miRNAs, 23 miRNAs were significantly expressed under HS. Eleven differentially expressed miRNAs (DE miRNAs) and 9 their predicted targets were confirmed by qPCR, which were expected miRNA–mRNA negative regulation pattern. Among the DE miRNAs and targets, miR-141-39 may target *COQ6*, miR-449a-5p and miR-34c-5p may control *RFC5* and *RTN2* together, miR-449a-5p may target *ACADVL*, miR-34c-5p potentially targets *Bcl-2* and miR-196b-5p potentially regulates *CASK* and *HOXB6*. Thus, the current work suggested the negative effects of HS on the ovarian tissue of female rabbits, and in conclusion these changes could be caused by decreased serum *IL-2, IL-8, CAT* and *GSH-Px* levels, increased ovarian apoptosis, and changed the expression of miRNAs.

## Introduction

The anatomical description of ovaries is characterized by the oval shape of glandular structures responsible for the production of estrogen and progesterone (1). Moreover, they play a vital role in regulating the menstrual cycle and conception *via* ovulation (2). The physiology of ovary is governed by ovarian internal factors and gonadotropins; together, they regulate the follicular microenvironment (3).

Animal husbandry is severely affected due to the effect of HS (4), leading to a series of economic losses as well as compromised animal health (5). HS could have deleterious effects on the immune and oxidative status of animals as well as on ovarian tissue (6). According to previous studies, *IL-8* and *IL-2* as cytokines are closely related to immune function, and *CAT* and *GSH-Px* as antioxidase seriously affect the body's antioxidant function (7–9). Meanwhile, a literature review revealed studies reporting impaired development of ovarian follicles upon exposure to HS and ultimately affecting ovarian function (10). Moreover, HS could affect follicle development and negatively affect reproduction by inducing autophagy and apoptosis in pig ovaries (11). In rabbits, with prolonged exposure to elevated ambient temperatures, HS and concomitant oxidative stress could be observed, impairing physiological functions, and high temperature could impair viability, feed consumption, growth, and fecundity by affecting endocrine mechanisms (12, 13). Hence, it is greatly necessary to understand the underlying mechanisms of HS-induced altered ovarian function. Furthermore, due to similarities in ovulation and conception, a rabbit model is the most suitable to study the effects of HS on ovarian function (14).

miRNAs are small non-coding RNAs that limit the translation of target mRNAs by binding to the RNA-induced silencing complex (RISC) (15). It plays an important role in the regulation of cellular events such as ontogenesis, cell proliferation, differentiation, occurrence, and development of tumors as well as antiviral action (16). Meanwhile, it is also involved in the cellular pathways of the complex RNA network (17). Additionally, owing to their different expression levels in ovarian tissues, the miRNAs could involve in follicular development, proliferation, and differentiation of follicles as well as ovulation by targeting several genes (18). What's more, miRNA pathways may be involved in HS, and some potential approaches could be provided to counteract the negative effects of HS on animals (19). The research shown that the recent trends employed in research involving the study of physiological effects of HS make use of small RNA-seq in the analysis of gene expression (20). However, no studies have yet reported miRNA expression profiles in HS-induced alterations in the ovarian tissue in rabbits (21). Therefore, the current work aims to evaluate changes associated with HS-induced physiological function and ovarian tissue in female rabbits.

## Materials and methods

### Experimental design, animals, and housing

Young, healthy and virgin female New Zealand white rabbits (*n* = 46) aged 7~8 months were provided by the Sichuan Academy of Animal Science. All rabbits were preconditioned for 2 weeks in a constant temperature room at 23°C. After that, these rabbits were randomly divided into two groups including HS group (23 rabbits) and CON group (23 rabbits). Each rabbit was kept in an individual cage during the experiment and provided with food and water. During the experiment, the daily temperature and humidity were recorded at pre-specified time points (9:30, 12:00, and 17:30) by an electronic hygrograph (Delixi, Jinhua, Zhejiang, China). The rabbits in the CON group were placed in an air-conditioned room at 23°C with a relative humidity of 85%. Similarly, rabbits in the HS group were placed in the room used electrical heater at 31°C with a relative humidity of 75% for 9 h daily (9:00–18:00) and 15 days. The temperature-humidity index (THI) was calculated by THI =*t* –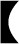
(0.31–0.31RH)^*^ (*t* – 14.4)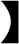, where “RH” is relative humidity/100 and “*t*” is ambient temperature (22). Therefore, the THI of CON group is 23.4 ± 0.4 and the THI of HS group is 30.05 ± 0.18. The initial and final body weight of each rabbit was recorded along with its daily food intake. Gross body weight gained and daily weight gained were calculated for each female rabbit according to the equations developed by Mutwedu et al. (13), as body weight gain = final body weight-initial body weight; daily weight gain = body weight gain/period (15 days).

### Sample collection

The rectal temperature of all rabbits was measured within the last 3 days of the experimental protocol (once a day). At the end of the experimental period, the blood samples from all rabbits were collected and stored appropriately until further analysis. Furthermore, a total of three rabbits were selected randomly from each group and sacrificed using euthanasia by injecting 20~30 ml of air into the ear vein to obtain tissue/organ samples. The collected ovaries were weighed subsequently to obtain gross weights. Relative ovary weight was calculated by: relative ovary weight = bilateral ovary weight/body weight. All ovary samples were segregated into two halves. The partial ovarian sample was fixed in 4% paraformaldehyde until histological analysis, whereas another ovarian sample was cryopreserved at liquid nitrogen until further miRNA analysis.

### Serum biochemical level analysis

Serum samples (*n* = 6) were analyzed for the concentrations of several related oxidative biomarkers including Catalase (*CAT*), Glutathione peroxidase (*GSH-Px*), Interleukin 2 (*IL-2*), and Interleukin 8 (*IL-8*) using the ELISA technique (23). The serum levels were measured according to the instructions provided by the manufacturer using *CAT, GSH-Px, IL-2*, and *IL-8* ELISA kits (R&D Systems, Minneapolis, MN, United States). The detection sensitivity was <1.0 U/ml, 1.0 U/ml, 1.0 pg/ml, and 1.0 pg/ml, respectively. The inter-assay and intra-assay coefficients of variation were both <15%. The *R* values of the respective standard linear regression and expected concentration correlation coefficients were all ≥0.9900.

### Histological assay

Histological analysis was performed on multiple ovarian tissues (*n* = 6) fixed with 4% paraformaldehyde (Fuchen Chemical Reagent Co., Ltd, Tianjin, China). The tissue samples were dehydrated by xylene and ethanol (75, 85, 95, and 100%; Haixing Chemical Reagent Factory, Chengdu, China), further embedded in paraffin wax, and sliced into 5 μm thickness. These thin sections were then stained using Hematoxylin-eosin (H&E; Sewell Biotechnology Co., Ltd, Wuhan, China) and observed under a light microscope (Nikon, Japan) (24). Ovarian follicles were generally classified into primordial follicles, primary follicles, secondary follicles and mature follicles. At the same time, we counted the number of follicles and vacuolated follicles in each stage of the ovary.

### TUNEL assay

The apoptotic cell within the ovary sample was analyzed by TUNEL assay (25). The samples were prepared using the In Situ Cell Death Detection Kit according to the instructions provided by the manufacturer (Roche, Shanghai, China). In brief, the tissue samples were washed with xylene and ethanol (Fuchen Chemical Reagent Co., Ltd, Tianjin, China) and embedded in paraffin wax which was sliced into 5 μm sections. These thin sections were incubated with citrate repair solution for 8 min and the TUNEL reaction mixture for 1 h at 37 °C and maintained under dark conditions. In the final step, DAPI (Servicebio Biotech, Wuhan, China) was added to the sections and incubated for 10 min. Apoptotic cells were analyzed by fluorescence microscopy (Leica DMI4000B, Wetzlar, Germany). At least nine images were collected for each repetition. TUNEL positive cells were observed and counted under a fluorescence microscope, and the apoptosis rate was counted and analyzed by using Image J. Cells apoptosis rate = TUNE positive cells/DAPI cells.

### RNA extraction, small RNA library construction, and sequencing

Total RNA isolation was performed using TRIzol Reagent (Takara, Dalian, China) following the manufacturer's protocol from the six ovary samples. RNA concentration, purity, and integrity were measured using the Nanophotometer^®^ spectrophotometer (IMPLEN, Westlake Village, CA, USA) and the Agilent 2100 Bioanalyzer (Agilent Technologies, CA, USA), with RNA quality ranging from 1.8 to 2 and RNA integrity number from 9.0 to 10.0.

Furthermore, six small RNA cDNA libraries were generated using the NEBNext^®^ Multiplex Small RNA Library Prep Set for Illumina^®^ (NEB, USA) as per the manufacturer's instruction and further sequenced on an Illumina SE50 platform generating 50-bp single-end (SE) reads. After screening the low-quality sequences, the clean reads of lengths 18–35 nucleotides (nt) were mapped to the rabbit reference genome and analyzed using Bowtie2 (v2.2.9) combined with the Rfam database (26). The known miRNAs alignment and prediction of novel miRNAs were based on the miRbase v22 database (http://www.mirbase.org/) and miRDeep2 software (27). Moreover, DE miRNAs expression levels were estimated by TPM (transcript per million) through the following criteria of |log_2_FC| ≥ 1 and corrected *P*-value < 0.05. Furthermore, the DE miRNAs analysis in two groups was performed using the DESeq R package (1.24.0). The target genes of DE miRNAs were identified by miRanda and RNAhybrid for rabbits with the reference genome. The overlapping genes were further used for GO and KEGG analysis. GOseq was implemented for GO enrichment analysis. The KEGG annotations were made through the online KEGG database (https://www.kegg.jp/kegg/kegg1.html) (28). We used KOBAS software to test the statistical enrichment of the target gene candidates in KEGG pathways (29).

### Validation of small RNA sequencing data and target genes by QPCR

The reliability of DE miRNAs obtained from small RNA sequencing and genes were confirmed by choosing DE miRNAs and their target genes for qPCR validation. Moreover, miRNA cDNA was synthesized from total RNA using the Mir-X™ miRNA First-Strand Synthesis Kit (Takara Biotechnology Co., Ltd., Dalian, China) and total RNA were reversed by using the PrimeScript™ RT reagent Kit with gDNA Eraser (Takara Biotechnology Co., Ltd., Dalian, China) according to the manufacture's instruction. Furthermore, the quantification of miRNAs and mRNA was performed on the Bio-Rad CFX96 RT-PCR detection system (Bio-Rad, USA). PCR was done using a final volume of 10 μl/well, including 5 μl SYBR Green Super Mix (Bio-Rad, Hercules, CA, USA), 1 μl template cDNA, 0.4 μl of each primer (10 pmol/μl), and 3.2 μl of RNAse-free water. Reaction conditions were 95.0°C for 10 s, 39 cycles of 95.0°C for 5 s and 60.0°C for 20 s, subsequently 95.0°C for 1 min, then Melt Curve from 65 to 95°C per increment 0.5°C for 5 s to read the plate. U6 and GAPDH were used as internal reference genes. Relative expression levels of miRNAs and genes were normalized by the method of 2^−Δ*ΔCT*^ (30). The RT-qPCR primers of selected genes and miRNAs were listed in Supplementary Table S1.

### Statistical analysis

All results were expressed as mean ± SEM. All data were tested for normal distribution and *t*-test using SPSS 26 and Prism 8 software (GraphPad, San Diego, CA, United States). *P* < 0.05 was considered statistically significant. Moreover, * denotes *P* < 0.05, ** denotes *P* < 0.01, *** denotes *P* < 0.001 and **** denotes *P* < 0.0001.

## Results

### Effect of HS on the growth performances of rabbit

**Figure 1:**
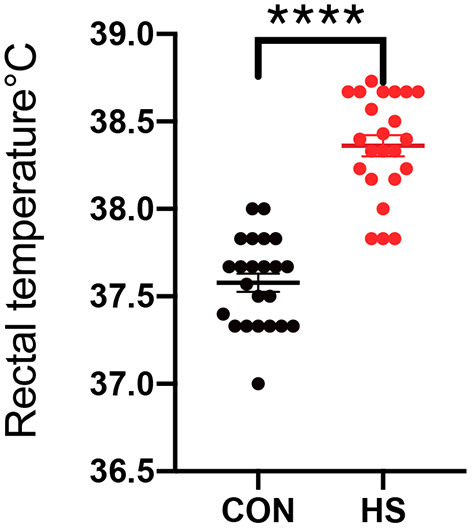
Rectal temperature of female rabbits (*n* = 23 per group) in HS group and CON group. CON indicates CON group and HS indicates HS group. ****indicated statistically significant difference.

The HS status of female rabbits was evaluated by analysis of gross examinations such as weight gain, rectal temperature and ovary weight. As shown in Table 1 and Figure 1, body weight gain was significantly lower, and the rectal temperature was significantly higher in the HS group in comparison with the CON group. In Table 2, the relative weight of ovaries showed a significant reduction after HS treatment in comparison to the CON group.

**Table 1:** Growth performances for female rabbits (*n* = 23 per group) in 15 days, as affected by HS.

**Items**	**CON (*n* = 23)**	**HS (*n* = 23)**	***P*-value**
Initial body weight (g)	4,212.3 ± 63.96	4,231.65 ± 61.69	0.8287
Final body weight (g)	4,340 ± 70.72	4,268.3 ± 66.3	0.4634
Average daily intake (g)	164.67 ± 6.37	160.33 ± 5.85	0.618
Body weight gain (g)	127.7 ± 26.98	36.65 ± 24.78	0.0168^*^
Daily weight gain (g)	8.51 ± 1.8	2.44 ± 1.65	0.0168^*^

Body weight gain = final body weight – initial body weight; daily weight gain = body weight gain/period (15 days).

* Indicated statistically significant difference.

**Table 2:** Relative ovary weight for female rabbits (*n* = 3 per group), as affected by HS.

**Items**	**CON (*n* = 3)**	**HS (*n* = 3)**	***P*-value**
Bilateral ovary weight (g)	0.44 ± 0.02	0.30 ± 0.02	0.0061^**^
Relative ovary weight (g/kg)	0.109 ± 0.007	0.071 ± 0.003	0.0108^*^

Relative ovary weight = bilateral ovary weight/body weight.

* and ** Indicated statistically significant difference.

### Effect of HS on rabbit serum biomarkers

**Figure 2:**
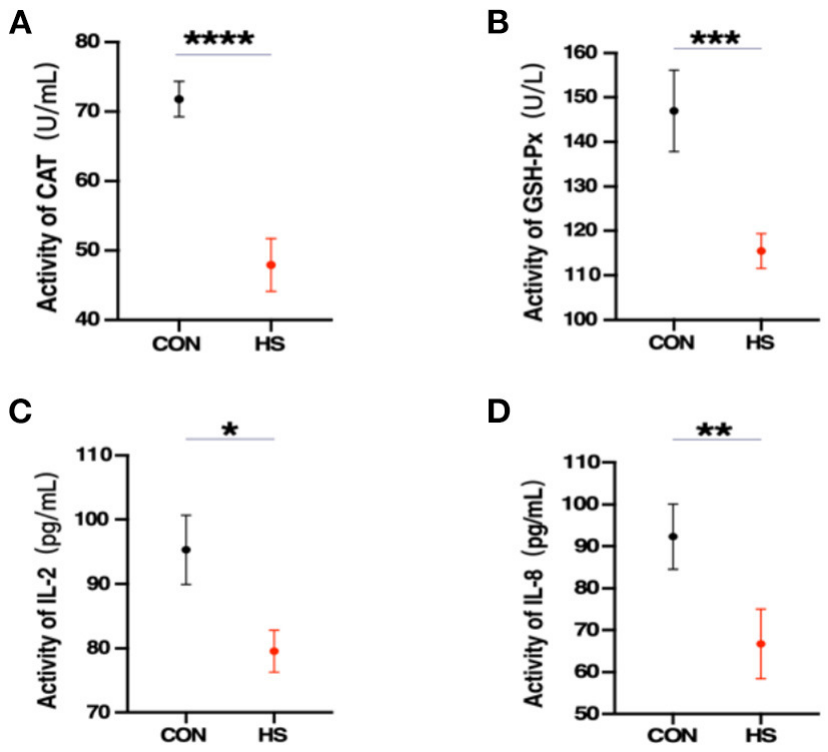
**(A–D)** Serum oxidative and immune biomarkers levels (*n* = 3 per group) from the HS group and CON group. *CAT*, catalase; *GSH-Px*, glutathione peroxidase; *IL-2*, interleukin 2; *IL-8*, interleukin 8. *, **, *** and **** indicated statistically significant difference.

Furthermore, the physiological state of rabbit can be observed by measuring changes in serum concentrations. As shown in Figure 2, significant alterations were observed in the serum levels of several related oxidative and immune biomarkers including *CAT, GSH-Px, IL-2*, and *IL-8*. This suggests that HS caused a decrease of immunity and antioxidant capacity in female rabbits.

### Effect of HS on histomorphology and apoptosis of rabbit ovary tissue

**Figure 3:**
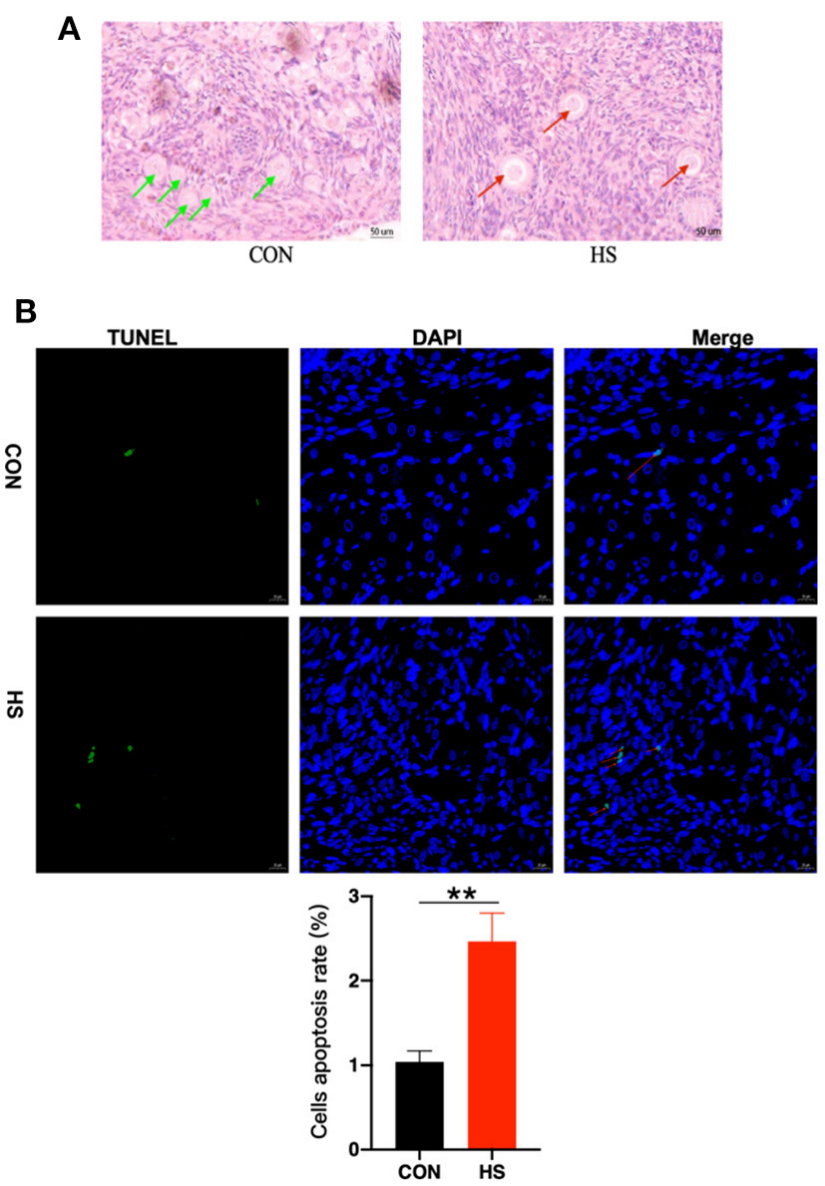
**(A)** Ovarian histomorphology (*n* = 3 per group) in HS group and CON group. Green arrows indicated normal primary follicles and red arrows indicated vacuolated primary follicles; **(B)** Apoptotic cell count and cells apoptosis rate in ovarian tissues (*n* = 3 per group) in HS group and CON group. Nuclei were stained with blue dye (DAPI). Furthermore, red arrows indicated TUNEL-positive cells (green, Bar = 20 μm). Cells apoptosis rate = TUNEL positive cells/DAPI cells. ** indicated statistically significant difference.

Additionally, gross pathological examinations were also made to determine the effect of HS on ovarian tissue. Parameters such as histopathological analysis and TUNEL validation of rabbit ovaries were employed. As shown in Figure 3A. the incidence of vacuolization of follicles was observed upon HE staining after HS treatment, especially in the primordial follicles. Meanwhile, in Table 3, there was no difference in the number of ovarian follicles between HS and CON, but the number of vacuolated follicles was significantly higher in the HS group. In addition, the results of the TUNEL assay revealed significant enhancement in cellular apoptosis in ovarian tissues of the HS group as compared to the CON group, as shown in Figure 3B.

**Table 3:** The number of follicles in various stages for the ovary of female rabbits (*n* = 3 per group), as affected by HS.

**Items**	**CON (*n* = 3)**	**HS (*n* = 3)**	***P*-value**
Primordial follicle	202.67 ± 20.83	210 ± 5.29	0.7501
Primary follicle	11.67 ± 1.76	13.33 ± 4.33	0.7397
Secondary follicle	6.33 ± 1.2	7.33 ± 0.67	0.5072
Mature follicle	4.33 ± 0.67	3 ± 1.15	0.3739
Total follicle	225 ± 20.5	233.67 ± 7.84	0.7131
Vacuolated follicle	11 ± 3.61	36.33 ± 4.41	0.011^*^

* Indicated statistically significant difference.

### Effect of HS on miRNA expression profile in ovarian tissue

**Figure 4:**
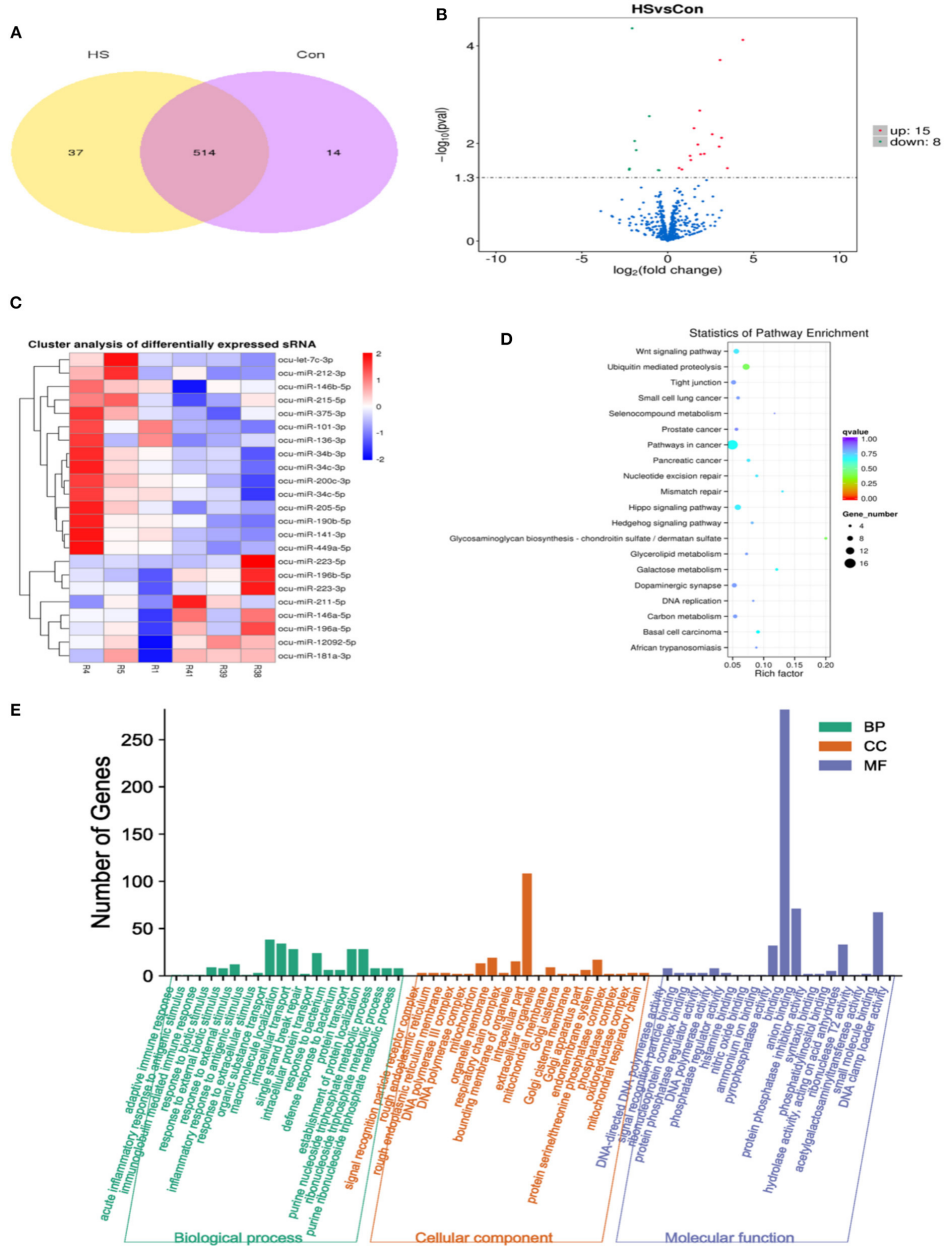
Analysis of DE miRNAs from rabbit ovary in CON group and HS group. **(A)** Venn diagram illustrating common and different DE miRNAs between HS and CON; **(B)** Analysis of DE miRNAs by volcano map; **(C)** Hierarchical cluster diagram of DE miRNAs; **(D)** KEGG pathway enrichment analysis of target genes in DE miRNAs; **(E)** GO enrichment analysis of target genes in DE miRNAs.

The miRNA expression profiles of rabbit ovaries were analyzed by small RNA sequencing in the HS group. After an initial screening of the raw data, a total of 60,393,038 high-quality reads were obtained from six different libraries, as given in Supplementary Table S2 (R1, R4, and R5 are HS group; R38, R39, and R41 are CON group). Furthermore, the sequence length analysis of the identified miRNAs found that most miRNAs were between 20 and 24 nt in length, with 22 nt RNAs being the most abundant. Moreover, two known miRNAs 451 and 471, as well as two novel miRNAs 77 and 80 were identified in the CON and HS groups, respectively in Supplementary Table S4. Additionally, common 514 miRNAs including known miRNAs 442 and novel miRNAs 72 were identified in both the HS group and CON group, as depicted in Figure 4A. Meanwhile, the entire information of miRNAs (known and novel miRNAs with TMP) was shown in Supplementary Table S5. Moreover, the hierarchical clustering analysis revealed that the expression profiles of the DE miRNAs showed significant changes in the HS group as compared to the CON group, as depicted in Figure 4C. Furthermore, these DE miRNAs (*n* = 23) were screened with volcano plots, which revealed up-regulated miRNAs (*n* = 15) and down-regulated miRNAs (*n* = 8), as shown in Figure 4B and Supplementary Table S3.

Meanwhile, to investigate the biological functions involved in the regulation of DE miRNAs, GO enrichment analysis was employed on the predicted target genes of these DE miRNAs. Moreover, as depicted in Figure 4E, the 570 predicted target genes were annotated into three main GO categories, including biological processes, cellular component and molecular functions. For biological processes, most genes were enriched in organic substance transport, macromolecule localization, intracellular transport, protein transport, establishment of protein localization, response to external stimulus, response to external biotic stimulus and response to biotic stimulus. For cellular component, most enriched terms included intracellular part and organelle membrane. For molecular function, most enriched terms included binding, anion binding and small molecule binding. Additionally, as shown in Figure 4D, KEGG enrichment analysis of the predicted target genes of DE miRNAs showed that their targets were mainly enriched in the cancer cell signaling pathways, followed by the Wnt signaling, Hippo signaling, Hedgehog signaling and galactose metabolism.

### Analysis of selected miRNA–mRNA expression level

**Figure 5:**
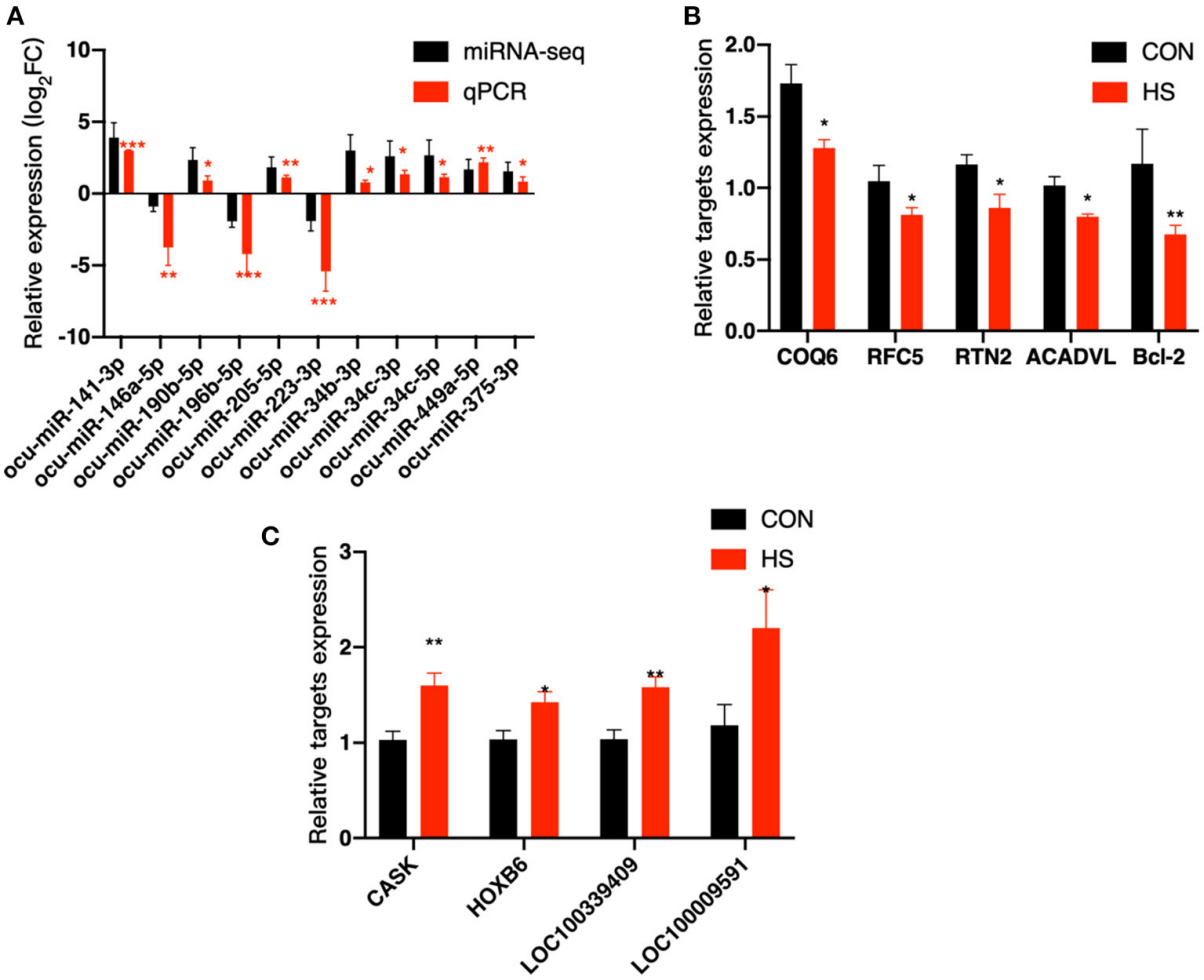
Validation of DE miRNAs and their targets was done by qPCR. **(A)** Validation of 11 DE miRNAs; **(B,C)** Validation of potential targets. *COQ6*, coenzyme Q6; monooxygenase; *RFC5*, replication factor C subunit 5; *RTN2*, reticulon 2; *ACADVL*, acyl-CoA dehydrogenase very long chain; *Bcl-2*, B-cell CLL/lymphoma 2 protein; *CASK*, calcium/calmodulin dependent serine protein kinase; *HOXB6*, homeobox B6; *LOC100339409*, ATPase inhibitor; mitochondrial-like; *LOC100009591*, sodium channel protein type 1 subunit alpha. * and ** indicated statistically significant difference.

From DE miRNAs screened by small RNA-Seq, 11 miRNAs (miR-141–3p, miR-146a-5p, miR-223–3p, miR-190b-5p, miR-196b-5p, miR-205–5p, miR-34b-3p, miR-34c-3p, miR-34c-5p, miR-449a-5p, and miR-375–3p) were randomly selected for qPCR validation. As shown in Figure 5A, the results of miRNA analysis obtained from qPCR and miRNA-seq indicated that the trend is consistent with change in expression profiles, therefore, highlighting the reliability of the sequencing results. Meanwhile, nine potential genes were selected from 11 DE miRNAs targets for qPCR validation (Supplementary Table S6), including COQ6, RFC5, RNT2, Bcl-2, ACADNL, CASK, HOXB6, LOC100339409 and LOC100009591. The qPCR results of our targets were consistent with the expected miRNA-mRNA negative regulation pattern. The results showed that miR-141-3p, miR-449a-5p and miR-34c-5p were significantly up-regulated upon HS and that their predicted target genes COQ6, RFC5, RTN2, Bcl-2 and ACADVL were significantly down-regulated during HS, as depicted in Figure 5B. A significant decrease in the expression of miR-196b-5p and miR-223-3p was observed upon HS, and the predicted target genes of those miRNAs, CASK, HOXB6, LOC100339409 and LOC100009591 were significantly up-regulated during HS, as depicted in Figure 5C.

## Discussion

HS could lead to huge economic losses in animal husbandry by reducing animal reproductive performance (12, 31). It has been reported that the rectal temperature and daily weight variations are considered good indicators of HS status (32). Furthermore, similar findings were observed in rabbits from the HS group, revealing a significant increase in rectal temperature and a decrease in body weight, indicating that the female rabbit was in a state of severe stress (13, 33). A significant reduction in the relative weight of the ovaries 15 days of HS exposure in rabbits was observed, which was consistent with the results of previous studies, indicating that HS could induce impaired ovarian responsiveness (13, 34).

In the current research, the serum *CAT* and *GSH-Px* were significantly downregulated, indicating HS impaired the rabbit's antioxidant capacity. Previous reports have demonstrated that HS induces oxidative stress and reduces antioxidant defenses in animals including decreased *CAT* and *GSH-Px* activity (35). Many studies have reported that *CAT* and *GSH-Px* functions as antioxidase to get rid of lipid oxides produced during cell metabolism, thus, preventing peroxide poisoning (7). What's more, *CAT* and *GSH-Px* could act as major antioxidant regulators in the initial stage of folliculogenesis (36, 37). In addition, oxidative stress, caused by an imbalance between the generation and elimination of intracellular reactive oxygen species (ROS), directly disrupts the internal ovarian environment, which reduces the communication between oocytes and granulosa cells (GCs) and eventually affects the maturation of oocytes before ovulation (38). Previous studies have shown that the accumulation of ROS in the ovaries affects folliculogenesis and ovulation, and ultimately leads to ovarian aging (38, 39). Meanwhile, the current work reported that HS could influence the inflammatory response in female rabbits. Previous studies have reported the immuno-suppressive action of HS in animals (40). Pro-inflammatory cytokines such as *IL-2* and *IL-8* directly promote the inflammatory response against HS *via* activation, growth, and differentiation of T cell subsets, B lymphocytes, and NK cells (8, 9, 41, 42). In the present study, a significant decrease in the production of *IL-2* and *IL-8* in serum was observed, which could be corroborated by the decline in T lymphocyte function as a result of impaired immunity (43). Previous reports have demonstrated that HS induces an immune response and significantly reduces *IL-8* production (44). Meanwhile, changes in the immune response, including a significant reduction in the concentration of *IL-2*, were also observed in HS-treated broilers (45). In addition, *IL-2* and *IL-8* could play an important role in folliculogenesis, such as timely follicular rupture and ovulation (46–48). It has been shown that complex interactions between inflammatory factors and other factors mediate oocyte-GCs crosstalk and dysregulation of pro-inflammatory cytokines leads to impaired immunity, which ultimately affects folliculogenesis (49, 50).

In the current work, HE staining of the ovaries revealed undesirable and severe vacuolation of follicles. According to previous studies, the development of ovarian follicles is associated with and directly proportional to the reproduction function of the animal (51). We also found that the number of follicles at each stage appeared to be no difference, yet qualitatively HS significantly increased the amount of vacuolated follicles, especially primordial follicles. Consequently, follicular vacuolation could affect the quality of the developed follicles, leading to a surge in unhealthy follicles, and may promote the development of follicular atresia (11). Previous studies have highlighted that HS affects the primordial or primary follicles in the ovary, then this could reduce the amount of healthy follicles in the follicular reserve, thereby reducing the animal's lifetime reproductive performance (52). Thus, the current work revealed the effect of HS on ovarian follicle development in rabbits accounted by HE staining and follicle count. Furthermore, the apoptosis rate in ovarian cells was significantly increased. In addition, in nuclear apoptosis, which often represents the late stage of apoptosis, the chromosomal DNA of ovarian cells will be broken, resulting in a large number of sticky 3-OH ends, which will be marked after using TUNEL (53). It was reported that apoptosis plays an important role in germ cell depletion in the mammalian ovary and the apoptotic process impairs follicle development (38). However, whether the vacuolated primordial follicles recover from the effects of HS as the follicle develops awaits further investigations.

Furthermore, small RNA sequencing was employed to reveal the underlying molecular mechanisms in the ovary. Moreover, the current work reported previously unexplored effects of HS on the changes of miRNA expression profiles in the rabbit ovaries, indicating these DE miRNAs may be involved in female rabbit reproduction performance through changes in inflammation, oxidative stress and apoptosis. In general, 11 miRNAs were statistically different between the HS and CON groups, of which eight miRNAs (miR-141–3p, miR-190b-5p, miR-205–5p, miR-34b-3p, miR-34c-3p, miR-34c-5p, miR-449a-5p, and miR-375–3p) significantly increased and three miRNAs (miR-146a-5p, miR-223–3p, and miR-196b-5p) significantly decreased, which was consistent with the qPCR results. Among the up-regulated of miRNAs, miR-141-3p has been shown to play an important role by inducing apoptosis, oxidative stress and mitochondrial dysfunction, and regulate the occurrence of ovarian cancer (54, 55). miR-449a-5p could control the inflammatory responses of recipient T cells and induce apoptosis by AKT signaling (56, 57). miR-34c-5p could promote GCs apoptosis and it has been shown to be closely related to inflammatory responses and oxidative stress (58, 59). On the other hand, HS down-regulated miRNAs. It has been reported that miR-196b-5p can regulate the oxidative stress, apoptosis, and steroid production pathway of GCs, thus promoting follicular development and maturation (60). The miR-223-3p could regulate pyroptosis through NLRP3-Caspase 1-GSDMD signal axis (61). These data suggest that five candidate miRNAs may be involved in the process of HS-stimulated ovarian tissue abnormalities by regulating ovarian cell inflammation, oxidative stress, and apoptosis (62, 63).

To further understand the potential function of the DE miRNAs, their predicted genes were annotated by KEGG and GO. These genes were identified by comparing the two groups, which can be involved in certain cancer pathways such as the Hippo signaling, Wnt signaling, and Hedgehog signaling pathway. Furthermore, these pathways are responsible for the activation of gene transcripts associated with oxidative stress, inflammation, and apoptosis, thereby indicating the importance of these miRNAs in HS-induced ovarian alterations in the rabbit model (64–67). Among the binding annotation, *COQ6* is an important component of the mitochondrial inner membrane, where it plays a role in electron transport, and it is reported that si-COQ6 could respiratory chain defects, increase ROS production, and induce apoptosis (68). *RFC5* is a small subunit of replication factor and has been reported to involve in cell cycle progression (69). It is reported that downregulation of *CASK* could activate apoptosis and induce autophagic death through the JNK/c-Jun signaling pathway (70). *HOXB6* has been reported to be associated with the occurrence of ovarian tumors (71). In the intracellular part annotation, it has been reported that *RTN2*, a member of the RTN protein family, is closely related to the functions of the endoplasmic reticulum, including protein processing and secretion, and ER-related pro-apoptotic mechanisms (72). For response to external stimulus annotation, *ACADVL*, as an acyl-CoA dehydrogenase very long-chain deficiency, is one of the enzymes required for β-oxidation, and mitochondrial β-oxidation can act as a brake to suppress host inflammatory responses *in vivo* during infection (73). When miRNAs were differentially expressed in HS-treated ovaries, their predicted targets were also differentially expressed, suggesting that miRNAs may be involved in this process, especially when DE miRNAs and their predicted target genes were in a miRNA-mRNA negative regulation pattern, as this is a typical mode observed between a miRNA and its target gene (74). As most of these predicted target genes were associated with the inflammatory, oxidative and apoptotic processes, combined with the qPCR results, we speculated miR-141-3p may target *COQ6*, miR-449a-5p and miR-34c-5p may co-regulated *RFC5* and *RTN2*, miR-449a-5p possibly targets *ACADVL* and miR-196b-5p potentially regulates *CASK* and *HOXB6* to regulate molecular mechanisms within the ovary during HS. In addition, it has been demonstrated in previous studies that miR-34c-5p could directly target Bcl-2 to regulate the apoptosis of GCs (58). Interestingly, there was also a potential negative regulatory relationship between miR-34c-5p and Bcl-2 in our study, suggesting that miR-34c-5p could regulate ovarian tissue apoptosis by targeting *Bcl-2*, which may serve as a therapeutic target in response to HS. In addition, the low expression of anti-apoptotic gene *Bcl-2* and the increase of TUNEL-positive cells in ovarian tissue under HS conditions suggest the occurrence of apoptosis. However, due to the limited sensitivity and specificity of TUNEL staining, further research is necessary (75). Undoubtedly, the regulatory relationship between miRNA and predicted target genes deserves further validation. However, the involvement of 5 candidate miRNAs in and mediating the effects of HS on the ovary awaits further validation.

## Conclusion

The current work concluded that HS decreases serum concentrations of *IL-2, IL-8, CAT*, and *GSH-Px*. Moreover, it also increased apoptosis in ovarian cells, causing a surge of vacuolated follicles. Additionally, it was also performed that the changes in miRNA expression profile in rabbit ovaries were associated with HS by small RNA-seq. Therefore, combined with qPCR validation, we speculated that 5 candidate miRNAs might be involved in the regulation of HS-stimulated ovarian abnormalities by targeting predicted target genes. Overall, the current work highlights and confirms the negative influence of HS on ovarian tissue, which may be regulated by candidate miRNAs as well as serum *IL-2, IL-8, CAT*, and *GSH-Px* levels. Candidate miRNAs involved in and mediating the effect of HS on ovarian tissue and the relationship between candidate miRNAs and their target genes await further validation.

## Data availability statement

The datasets presented in this study can be found in online repositories. The names of the repository/repositories and accession number(s) can be found in the article/Supplementary material.

## Ethics statement

The animal study was reviewed and approved by Ethical Standards of the Use Committee of the College of Animal Science and Technology, Sichuan Agricultural University, Sichuan, China (20220183). Written informed consent was obtained from the owners for the participation of their animals in this study.

## Author contributions

LT, XB, GC, XJ, XX, ML, CL, and SL participated in the conception and design of the experiment. LT, XB, and GC performed the experimental protocols. LT analyzed the experimental data and drafted the manuscript. SL revised the manuscript. All authors have read and approved the manuscript.

## Funding

The current study was funded by the National Modern Agricultural Industrial Technology System (CARS-43-A-2), the Key R&D Project of Sichuan Province (2021YFYZ0033), and the National Natural Science Foundation of China (32102530).

## Conflict of interest

The authors declare that the research was conducted in the absence of any commercial or financial relationships that could be construed as a potential conflict of interest.

## Publisher's note

All claims expressed in this article are solely those of the authors and do not necessarily represent those of their affiliated organizations, or those of the publisher, the editors and the reviewers. Any product that may be evaluated in this article, or claim that may be made by its manufacturer, is not guaranteed or endorsed by the publisher.
